# Evaluation of FDG-PET/CT Use in Children with Suspected Infection or Inflammation

**DOI:** 10.3390/diagnostics10090715

**Published:** 2020-09-18

**Authors:** Fabienne G. Ropers, Robin M. P. van Mossevelde, Chantal P. Bleeker-Rovers, Floris H. P. van Velden, Danielle M. E. van Assema, Judit A. Adam, Marnix G. E. H. Lam, Nelleke Tolboom, Olaf M. Dekkers, Lioe-Fee de Geus-Oei, Virginie Frings

**Affiliations:** 1 Department of Pediatrics, Leiden University Medical Center, Albinusdreef 2, 2333 ZA Leiden, The Netherlands; R.M.P.van_Mossevelde@lumc.nl (R.M.P.v.M.); V.Frings@lumc.nl (V.F.); 2 Department of Internal Medicine, Radboud University Medical Center, Geert Grooteplein Zuid 10, 6525 GA Nijmegen, The Netherlands; chantal.bleeker-rovers@radboudumc.nl; 3 Section of Nuclear Medicine, Department of Radiology, Leiden University Medical Center, Albinusdreef 2, 2333 ZA Leiden, The Netherlands; F.H.P.van_Velden@lumc.nl (F.H.P.v.V.); l.f.de_geus-oei@lumc.nl (L.-F.d.G.-O.); 4 Department of Radiology and Nuclear Medicine, Erasmus Medical Center, Dr. Molewaterplein 40, 3015 GD Rotterdam, The Netherlands; d.vanassema@erasmusmc.nl; 5 Department of Radiology and Nuclear Medicine, Amsterdam UMC, Meibergdreef 9, 1105 AZ Amsterdam, The Netherlands; j.a.adam@amc.uva.nl; 6 Department of Radiology and Nuclear Medicine, University Medical Center Utrecht, Heidelberglaan 100, 3584 CX Utrecht, The Netherlands; M.Lam@umcutrecht.nl (M.G.E.H.L.); n.tolboom@umcutrecht.nl (N.T.); 7 Department of Clinical Epidemiology and Endocrinology, Leiden University Medical Center, Albinusdreef 2, 2333 ZA Leiden, The Netherlands; o.m.dekkers@lumc.nl; 8 Biomedical Photonic Imaging Group, University of Twente, Drienerlolaan 5, 7522 NB Enschede, The Netherlands

**Keywords:** FDG-PET/CT, pediatrics, infection, inflammation, diagnostic value, diagnostic information

## Abstract

[^18^F]-FDG-PET/CT ([^18^F]-fluoro-deoxyglucose (FDG) positron emission tomography/computed tomography (PET/CT)) is increasingly used as a diagnostic tool in suspected infectious or inflammatory conditions. Studies on the value of FDG-PET/CT in children are scarce. This study assesses the role of FDG-PET/CT in suspected infection or inflammation in children. In this multicenter cohort study, 64 scans in 59 children with suspected infection or inflammation were selected from 452 pediatric FDG-PET/CT scans, performed in five hospitals between January 2016 and August 2017. Main outcomes were diagnostic information provided by FDG-PET/CT for diagnostic scans and impact on clinical management for follow-up scans. Of these 64 scans, 50 were performed for primary diagnosis and 14 to monitor disease activity. Of the positive diagnostic scans, 23/27 (85%) contributed to establishing a diagnosis. Of the negative diagnostic scans, 8/21 (38%) contributed to the final diagnosis by narrowing the differential or by providing information on the disease manifestation. In all follow-up scans, FDG-PET/CT results guided management decisions. CRP was significantly higher in positive scans than in negative scans (*p* = 0.004). In 6% of diagnostic scans, relevant incidental findings were identified. In conclusion, FDG-PET/CT performed in children with suspected infection or inflammation resulted in information that contributed to the final diagnosis or helped to guide management decisions in the majority of cases. Prospective studies assessing the impact of FDG-PET/CT results on diagnosis and patient management using a structured diagnostic protocol are feasible and necessary.

## 1. Introduction

[^18^F]-fluoro-deoxyglucose (FDG) positron emission tomography/computed tomography (PET/CT) is increasingly used as a diagnostic tool in the pediatric population. In pediatric oncology, FDG-PET/CT is frequently deployed for diagnosis and therapy response evaluation [1]. For other indications, such as suspected infection or inflammation, this is not the case. In these patients, FDG-PET/CT might also be advantageous because of its ability to detect cells with elevated glucose metabolism, which is already increased in the very early stages of infection or inflammation. FDG-PET/CT shows higher sensitivity in early and low-grade infections compared to CT or magnetic resonance imaging (MRI) [2]. FDG-PET/CT, however, cannot distinguish between infectious, inflammatory or oncologic etiology, but if necessary can guide further diagnostic testing to differentiate them [3]. Especially in patients with a yet undefined source of infection or inflammation, a whole-body imaging technique might decrease the time to diagnosis and treatment. However, even if FDG-PET/CT is recommended or considered, it is usually preceded by extensive other conventional imaging [4].

Literature reporting test performance in children with infection or inflammation comprises mainly retrospective studies based on data from health records of children with fever of unknown origin (FUO) [5,6,7,8]. In adults, the literature also includes prospective studies on the performance of FDG-PET/CT for various inflammatory or infectious diseases, such as FUO, gram-positive bacteremia and vasculitis [9]. It is likely that test performance in infection/inflammation is at least comparable in children with the advantage of a lower frequency of incidental findings, e.g., due to a lower incidence of malignancies in the pediatric population [10]. There are no studies describing the current practice of FDG-PET/CT in children, although its use is rising [11].

To assess whether diagnostic tools deliver what clinicians expect, it is important to monitor their use and diagnostic yield. Here, we describe the use of FDG-PET/CT in children with suspected or proven infection or inflammation and estimate the diagnostic yield and consequences of the test on clinical management. We also propose how to fill the knowledge gap around performance of this imaging technique in children.

## 2. Patients and Methods

### 2.1. Patients

FDG-PET/CT scans performed between January 2016 and August 2017 in children (age 0–17 years) with (suspected) infection or inflammation in the differential diagnosis were included from five hospitals in the Netherlands (Leiden University Medical Center, Amsterdam University Medical Center, Erasmus University Medical Center, University Medical Center Utrecht and Alrijne Hospital Leiderdorp). Clinical data were collected from the electronic patient records. Negative scans with a follow-up of less than three months after FDG-PET/CT were excluded.

### 2.2. FDG-PET/CT Scanning Procedure

All FDG-PET/CT scans were performed and reconstructed according to the European Association of Nuclear Medicine (EANM) guidelines [12] on scanners that received EARL (EANM Research Ltd.) accreditation, i.e., Biograph mCT, mCT Flow, Biograph Horizon with TrueV (Siemens Healthineers, Erlangen, Germany), Vereos and Gemini TF64 (Philips Healthcare, Best, the Netherlands). Patients fasted for at least 4 h prior to the intravenous FDG administration, compliant with the pediatric FDG dosage card [13]. One site (LUMC) used a slightly lower FDG dosage scheme that was in accordance with the EARL dose optimization protocol [14].

### 2.3. Data Sources

Annual pediatric FDG-PET/CT data were provided by the Dutch National Health Care Institute [11]. Original FDG-PET/CT scan reports were collected from the participating hospitals and were extracted for descriptive analysis. From the included patients, clinical data from the electronic patient files including presenting symptoms, presence of fever in the week preceding FDG-PET/CT, medication, laboratory results, microbiological testing, histopathological results and preceding radiological imaging during diagnostic work-up were collected. In addition, differential diagnoses, as described in the patient record, and on the request and report form before and after scanning, were documented. FDG-PET/CT findings were compared to diagnostic information of previous diagnostic tests. Information on further tests and treatment after FDG-PET/CT and the final diagnosis were extracted from the electronic patient file. Data were coded and ethical approval for this study was granted by the ethics committee of Leiden University Medical Center (reference G17.075, 28/05/2018).

### 2.4. Diagnostic and Follow-Up Scans

FDG-PET/CT scans were classified as diagnostic scans if they were performed as part of the primary work-up in establishing a diagnosis. If more than one diagnostic scan was performed before the correct diagnosis could be established and the reported abnormalities were comparable in both scans, only the first scan was included for analysis. Scans were classified as follow-up scans if they were performed to evaluate treatment effect or recurrence of a known disease. Data extraction was performed by R.M.P.v.M., and subsequent classifications were independently performed by two reviewers (F.G.R, V.F.). Discrepancies were resolved through discussion among authors (F.G.R, L.-F.d.G.-O., R.M.P.v.M., V.F.).

### 2.5. Analysis

#### 2.5.1. Diagnostic Accuracy and Reference Standard

Scans were considered true positive if non-physiological FDG uptake was related to the final diagnosis (see below). If uptake was incorrectly interpreted to be related to the symptoms that led to FDG-PET/CT scanning, FDG uptake was scored as false positive. If uptake was correctly interpreted to be unrelated to the final diagnosis, it was scored as unspecific uptake or an incidental finding. Incidental findings were assessed for their clinical relevance.

Scans were considered negative if only physiological/unspecific uptake or an unrelated incidental finding was detected. Scans were classified as false negative if a focal infection, inflammatory process or neoplasm that can reasonably be detected with FDG-PET/CT scanning was diagnosed by alternative diagnostic procedures and probably already present (based on signs and symptoms) at the time of the FDG-PET/CT scan. If a final diagnosis was made after a negative scan result, but positive scan findings would have been unlikely for this disease, the FDG-PET/CT scans in these cases were regarded as true negative. Additionally, a normal FDG-PET/CT was considered as true negative if, in the follow-up period of 3 months, no signs or results contradictory to the FDG-PET/CT results were found. In case of suspected infection, a follow-up period of 1 month was regarded as reasonable, since any disease diagnosed after this period could very well be unrelated to the initial symptoms.

The final diagnosis was based on all information obtained during the diagnostic process including results from blood culture, tissue culture, biopsy, serology, imaging, autopsy or a combination of these.

#### 2.5.2. Scan Consequence

We documented diagnostic and treatment decisions in relation to FDG-PET/CT as reported in the patient records. In diagnostic scans, contribution to final diagnosis was assessed. True positive scans could contribute to a final diagnosis through targeted diagnostics or in combination with other diagnostic clues. True negative scans could contribute to a final diagnosis by narrowing the differential diagnosis or providing more information on a certain diagnosis, e.g., by excluding metastatic disease in bacteremia.

#### 2.5.3. Incidental Findings

Incidental findings were defined as either a significant area of abnormal FDG uptake or an abnormality on the CT component of the FDG-PET/CT scan that was not evident on previous imaging and that was considered unrelated to the clinical indication for imaging. Non-pathological anatomic variants, line or pacemaker locations, past surgical interventions or old injuries were not considered incidental findings. Incidental findings were defined as relevant if they had an impact on patient management or outcome.

#### 2.5.4. Statistical Analysis

For association between C-reactive protein (CRP) and positive FDG-PET/CT scans, the Mann–Whitney test was used. For analysis, the first scan in the inclusion period was used. For association between fever (temperature > 38.3 °C), antibiotic use and positive FDG-PET/CT scans, Chi-square test was used. Calculations were performed using SPSS Statistics version 25 (IBM Corp. in Armonk, NY, USA). A *p*-value < 0.05 was considered statistically significant.

## 3. Results

### 3.1. Selection Process

**Figure 1:**
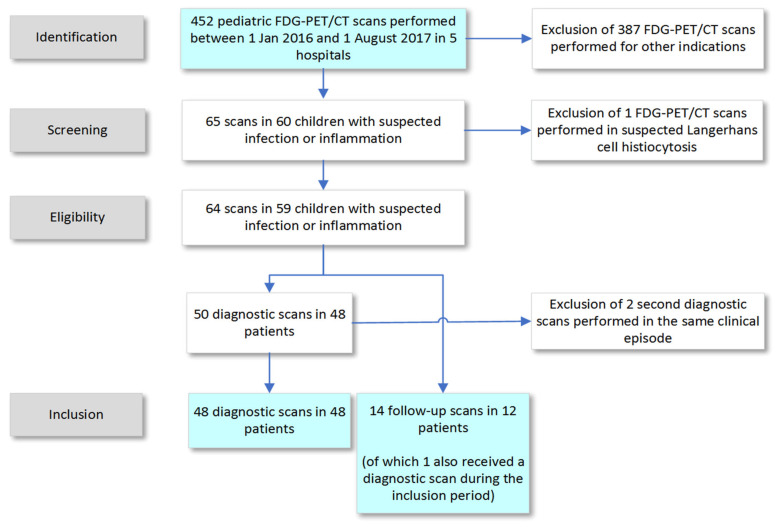
Selection of FDG PET/CT scans performed in five hospitals.

The annual number of total pediatric PET/CTs has gradually increased over recent years from 466 scans per year in 2012 to 666 scans per year in 2016 [11]. These numbers include PET/CTs with tracers other than FDG, but these constituted a minority. Between January 2016 and August 2017, 452 FDG-PET/CT scans with low dose CT scans for both attenuation correction and anatomical localization from the participating hospitals were considered (Figure 1). Sixty-five scans (14%) were included for further analysis, based on the presence of infection or inflammation in the differential diagnosis prior to FDG-PET/CT scanning. The majority of other FDG-PET/CT scans (81%) were performed for staging and therapy response monitoring in oncological disease, and another 7.5% in children with epilepsy. After a second revision of all scans, one scan was excluded because the provisional diagnosis was Langerhans cell histiocytosis, which was categorized as an oncological disease.

A total of 64 FDG-PET/CT scans performed in 59 patients (63% female) were included. The median age at first scan was 12 years (IQR 5–15 years, Table 1). Four patients received more than one scan, of which three patients had two scans during the inclusion period (two patients had two diagnostic scans, and one patient had two follow-up scans), and one patient had three scans (one diagnostic scan, two follow-up scans). Of these 64 scans, 50 were performed for primary diagnosis (diagnostic scans) and 14 were performed to monitor disease activity in children with established diagnoses (follow-up scans).

### 3.2. Diagnostic Scans

The diagnostic scans were performed in 48 patients; two patients received a second scan in the same diagnostic trajectory with comparable results. These second scans were excluded in the description below (Table 2).

**Figure 2:**
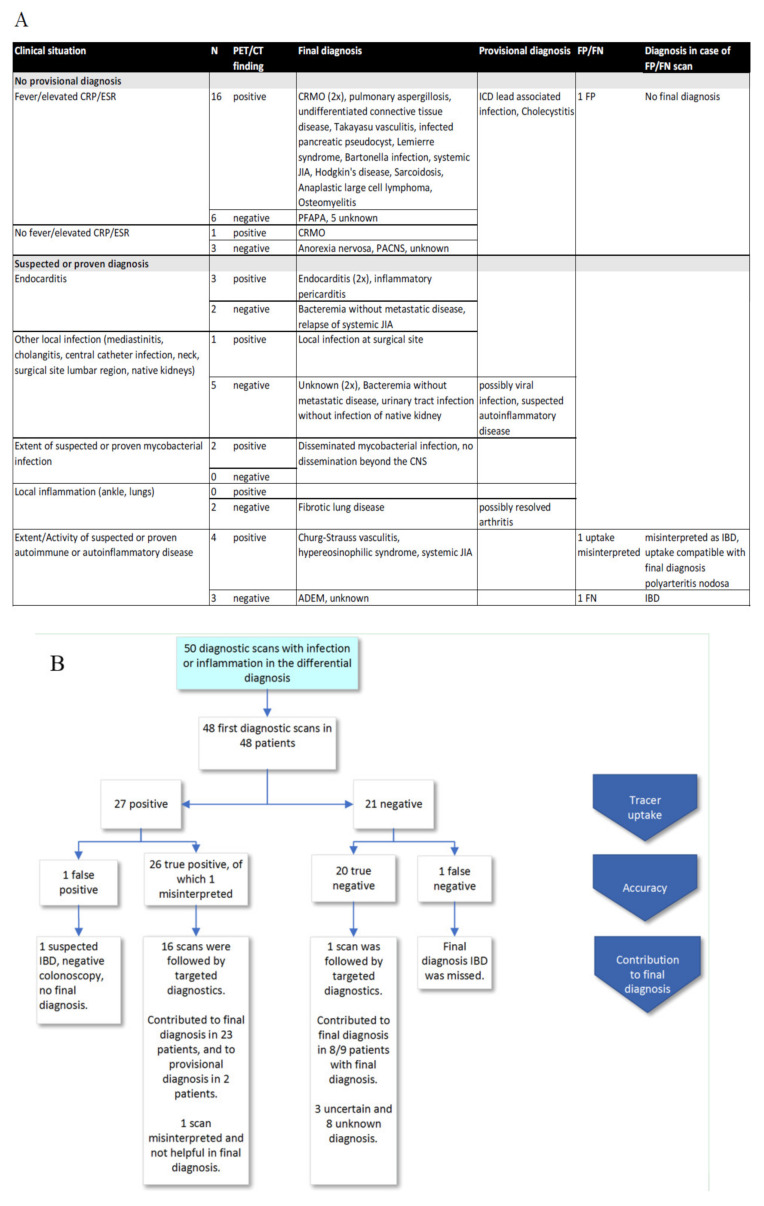
Scans performed for primary diagnosis (diagnostic scans). (**A**) Clinical situation, FDG-PET/CT findings and final diagnosis, false positive and negative findings. (**B**) Scan results, targeted diagnostics and contribution to final diagnosis. Abbreviations: ADEM: acute demyelinating encephalomyelitis, CNS: central nervous system, CRMO: chronic recurrent multifocal osteomyelitis, FN: false negative, FP: false positive, IBD: inflammatory bowel disease, ICD: implantable cardioverter-defibrillator, JIA: juvenile idiopathic arthritis, PACNS: primary angiitis of the CNS, PFAPA: periodic fever, aphthous stomatitis, pharyngitis, adenitis.

Of 48 diagnostic scans, FDG-PET/CT was requested in 26 cases in diagnostic situations with multiple diagnoses in the differential (Figure 2, and for detailed description of all scans in Supplementary Table S1). The differential included oncological, inflammatory and infectious conditions. Of these 26 scans without a provisional diagnosis, 22 were performed in the presence of unexplained fever or elevated inflammatory markers without localizing symptoms. In the other four cases, infection or inflammation was included in the differential diagnosis, based on clinical signs (unexplained joint pain, uveitis, fatigue, weight loss, or neurological symptoms). In 22 cases, FDG-PET/CT was performed to obtain specific information on established or suspected diagnoses (13 infectious, 9 inflammatory).

The 48 diagnostic scans were performed after a median of 41 days of symptoms (IQR 20–128 days). Extensive diagnostic examinations had already been undertaken before FDG-PET/CT in the majority of children (Table 2), and in 18/48 cases (38%), antibiotics had been administered the week before the FDG-PET/CT scan.

Of 48 diagnostic scans, 27 scans were positive, of which 26 were true positive (26/27, 96%) (Figure 2). In 26 true positive FDG-PET/CT scans, FDG-PET/CT revealed information that led to a provisional (*n* = 2) or final diagnosis (*n* = 23) either through targeted further evaluation (16 scans) or in conjunction with other diagnostic information (9 scans). In one patient with positive FDG-PET/CT, requested because of suspected large vessel vasculitis, FDG-PET/CT correctly ruled out large vessel vasculitis, but intestinal and lymph node uptake was interpreted to be indicative of inflammatory bowel disease (IBD). However, abdominal ultrasound, MRI, fecal calprotectin and colonoscopy were negative. Retrospectively, FDG-PET/CT findings were consistent with the final diagnosis of polyarteritis nodosa. The final diagnoses included infectious (*n* = 10), inflammatory (*n* = 12) and oncologic diseases (*n* = 2). In 1/27 scans, the outcome was false positive. In the false positive scan, intestinal uptake was suspicious of IBD but colonoscopy was negative and no final diagnosis was established.

Twenty-one scans were negative in nine patients with a broad differential before scanning and 12 with a provisional diagnosis. Of those, 20 were true negative. The specific diagnostic question was answered in 15/21 (71%) negative scans and a final diagnosis was established in 10/21 (48%) cases (three infectious, six inflammatory, one other (anorexia nervosa)). True negative scans contributed to the final diagnosis in 8/9 (89%) cases by narrowing down the broad differential diagnosis (2/9) or providing more information on a suspected diagnosis (6/9). The false negative scan concerned a patient with suspected chronic recurrent multifocal osteomyelitis (CRMO). FDG-PET/CT ruled out CRMO. The patient was ultimately diagnosed with IBD, but the FDG-PET/CT did not show increased intestinal uptake (false negative). In 11/21 (52%) cases, no certain diagnosis could be established (seven unknown, three probably resolved infection, one suspected autoinflammatory disease). 

In 34 of 48 diagnostic scans, a final diagnosis could be established (71%), of which in three cases FDG-PET/CT did not contribute to the final diagnosis. FDG-PET/CT findings revealed information leading to a diagnosis not previously considered in 4/34 (12%) of the cases with a final diagnosis. Of the positive scans, 23/27 (85%) were helpful in establishing a final diagnosis. Of the negative scans, 8/21 (38%) contributed to the final diagnosis by narrowing down the differential or providing information on a suspected diagnosis. In total, 31/48 (65%) scans seemed helpful in establishing a final diagnosis. Establishing a diagnosis had management consequences in all patients. In addition, in patients without a final diagnosis, management consequences of negative FDG-PET/CT findings were described, e.g., planning of cardiac surgery after exclusion of infection by FDG-PET/CT (Supplementary Table S1). However, in general it was difficult to specifically ascribe management to FDG-PET/CT results in diagnostic scans. Therefore, we do not present these findings in detail. In the majority of patients without a certain diagnosis, the symptoms disappeared.

### 3.3. Follow-Up Scans

**Figure 3:**
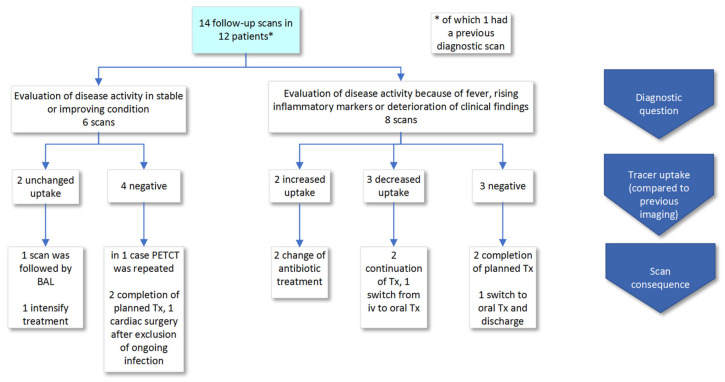
Scan results of FDG-PET/CT scans performed to monitor disease activity (follow-up scans). Abbreviations: Tx: treatment.

In 12 patients, 14 FDG-PET/CT follow-up scans were performed to monitor disease activity or therapy response (Figure 3). In 6/14 (43%) cases, FDG-PET/CT was performed in clinically stable or improving condition (a detailed description of all scans is in Supplementary Table S2). FDG-PET/CT was negative in four and showed unchanged uptake in two cases. In 8/14 (57%) scans performed because of suspected deterioration (persistent or recurrent fever, rising inflammatory markers or deterioration of clinical condition), the scans demonstrated increased uptake in two, decreased uptake in three and no uptake in three cases, in comparison to previous investigations. Because FDG-PET/CTs were requested to monitor disease activity, all scan results were used to inform treatment or other patient management decisions. There were no false negative or positive findings.

### 3.4. Relationship between CRP, Fever and Positive Scans

**Figure 4:**
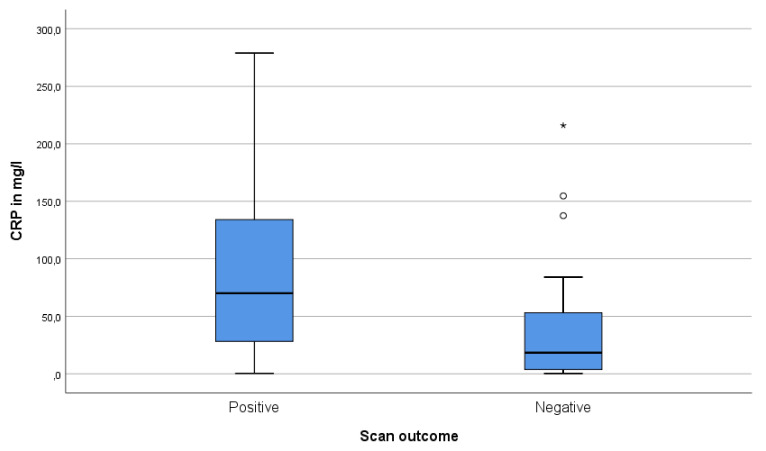
CRP measured less than a week before FDG-PET/CT scan in children with positive and FDG-PET/CT negative FDG-PET/CT scans (both first diagnostic and follow-up scans, *n* = 54 (59 patients, of which five were without documented CRP measurement the week before PET/CT scan; three in the positive scan group and two in the negative scan group). The bar indicates the median CRP, the box indicates the interquartile range (IQR), and whiskers indicate the range. Open circles represent outliers > 1.5 * IQR and an asterisk represents an outlier > 3 * IQR.

CRP in scans with positive findings was significantly higher than in negative scans (median 71 mg/L and 24 mg/L respectively, *p* = 0.004) (Figure 4). However, CRP distributions in the groups with positive and negative scan results were overlapping and low CRP did not preclude positive scans; 4/14 (29%) patients with CRP < 10 mg/L had positive scans. However, if CRP was > 10 mg/L, the majority of patients had positive scans (26/40, 65%). Fever and scan positivity were not associated (*p* = 0.56). In patients that received antibiotics in the week before scanning, 50% (9/18) had positive scans versus 60% (18/30) in those without antibiotics (*p* = 0.56).

### 3.5. Incidental Findings

In four cases (4/48, 8% of diagnostic scans), FDG-PET/CT revealed findings that were unrelated to the final diagnosis or the presenting complaint of the patient. Three of those (6%) showed findings with clinical relevance; one scan showed dislocation of a duodenal feeding tube that needed repositioning, one scan revealed fibrous dysplasia and one scan demonstrated an ectopic kidney.

### 3.6. Clinical Examples

**Figure 5:**
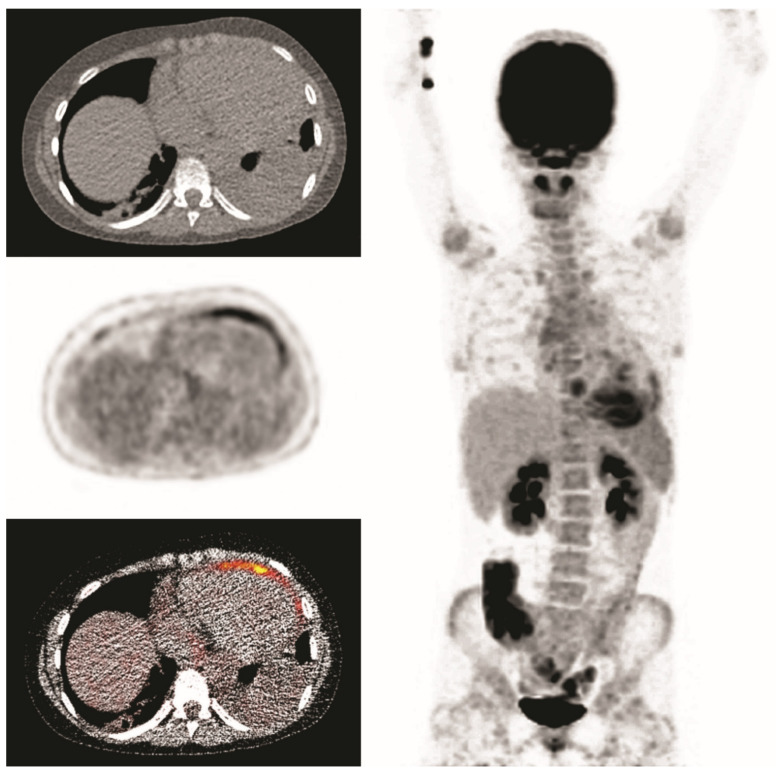
Clinical example 1. Pericardial FDG-uptake is present, most pronounced around the apex and anterior wall.

A 14-year-old girl, who previously underwent a mitral valve annuloplasty, was admitted with fever for one week with a maximum CRP of 290 mg/L. Echocardiography did not reveal a conclusive diagnosis. Endocarditis was suspected and FDG-PET/CT was performed after a low-carbohydrate diet. This revealed uptake in the pericardium but no signs of endocarditis (presented images are of first scan). She was treated with antibiotics for 6 weeks, and symptoms receded. However, microbiological testing remained negative. Six weeks after cessation of antibiotic treatment fever returned and a second FDG-PET/CT was performed. It showed similar pericardial uptake, suggestive for persisting pericarditis. She was successfully treated with colchicine, suggesting a non-infectious cause of pericarditis (Figure 5).

**Figure 6:**
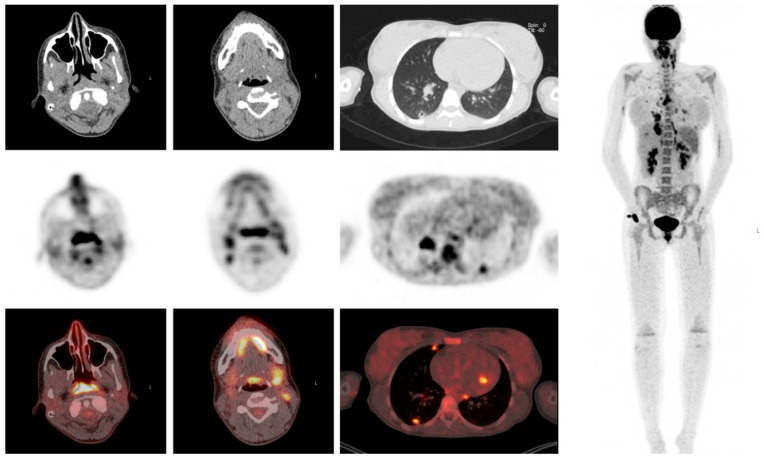
Clinical example 2. Intense FDG-uptake is visualized in the nasopharynx, tonsils, cervical and mediastinal lymph nodes and multiple pulmonary cavitating lung lesions.

A 16-year-old girl with an episode of sore throat one week earlier, presented with elevated inflammatory markers (maximum CRP 259 mg/L, ESR 60 mm/h), fever, hypotension and right upper abdominal pain radiating to the back. She was treated empirically with intravenous ceftriaxone. Because of persisting abdominal pain, ultrasound, chest X ray, and a diagnostic laparoscopy were performed. The latter because of suspected cholecystitis, which could be excluded. The fever subsided under antibiotic treatment. Because of persisting malaise and a yet unknown diagnosis, a FDG- PET/CT was performed. The differential diagnosis before FDG-PET/CT comprised infectious, autoimmune and oncologic causes. FDG-PET/CT revealed cavitating pulmonary lesions and FDG uptake in lymph nodes in the neck, mediastinum and abdomen and in the tonsils and nasopharyngeal region. FDG-PET/CT findings combined with the history of sore throat guided the diagnosis towards Lemierre syndrome, which was confirmed by ultrasound showing thrombosis of the jugular vein. After establishing the diagnosis and clinical improvement on IV antibiotics, the patient was discharged with oral antibiotics (Figure 6).

**Figure 7:**
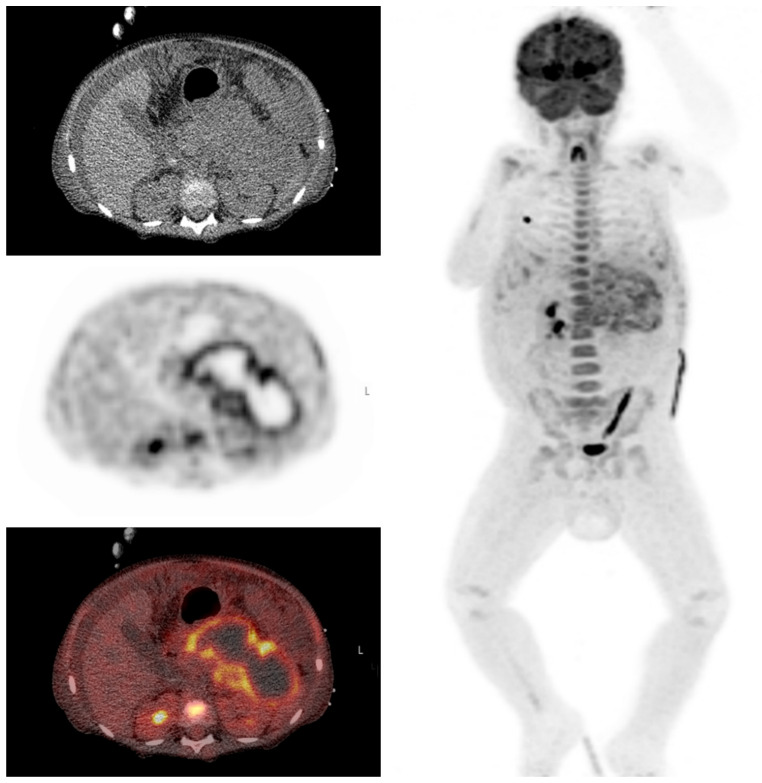
Clinical example 3. This scan shows an irregular hypodense lesion in the pancreatic region on low-dose CT and a photopenic area with hot rim on FDG-PET.

A 5-year-old boy with a history of nephrotic syndrome was admitted with fever, abdominal pain, nausea and a painful left leg. MRI of the leg did not show signs of osteomyelitis. CRP was 250 mg/L and pancreatic enzymes were elevated, suggesting pancreatitis. Antibiotics were started, but the fever persisted and his abdominal pain increased. An abdominal MRI was performed which showed signs of pancreatitis without cyst formation. A week later when symptoms were still persisting, FDG-PET/CT was performed, mainly to exclude other causes. FDG-PET/CT showed increased uptake in the periphery of an irregular hypodensity of 12 × 4 cm in the pancreas area, suggestive for an infected pancreatic pseudocyst. A transgastric drainage of the pseudocyst was performed and a stent was placed (Figure 7).

## 4. Discussion

This study describes the current use of FDG-PET/CT in the Dutch pediatric population, and included 64 scans from five different hospitals performed for suspected inflammatory or infectious conditions. These five hospitals performed approximately 50% of all FDG-PET/CT scans in children in the Netherlands [11], of which 14% concerned (suspected) infectious or inflammatory conditions. In the current study, FDG-PET/CT added information that was helpful in establishing the final diagnosis in 65% (31/48) of all diagnostic cases, of which 23/27 (85%) were positive and 8/21 (38%) were negative scans.

Elevated CRP level correlated with a positive FDG-PET/CT finding, although low CRP did not preclude positive findings. In a large cohort of children with FUO, Pijl et al. also found that CRP level was positively associated with true positive FDG-PET/CT [8].

Discriminating between physiologic and pathological uptake in the brain, heart, kidney and intestine can be challenging, and false positive findings are a well-described phenomenon [15,16]. Also in the present study, physiological intestinal tracer uptake led to misinterpretations; it led to futile additional testing, and, in one patient, IBD was overlooked. Overall, false positive and negative findings were rare, and in concordance with a previous study, relevant incidental findings in children were infrequent [10]. We did not present measures of diagnostic accuracy because sensitivity and specificity can vary over different suspected diagnoses, as does prior probability and thus predictive value. By including five hospitals, we captured 50% of all FDG-PET/CT scans performed in children. Because FDG-PET/CTs for oncological indications are mainly performed in academic centers, this could lead to an underestimation of the overall proportion of infectious or inflammatory indications. However, the number of scans performed outside academic centers could be small, because FDG-PET/CT is generally performed late in the diagnostic trajectory for suspected infection or inflammation, and patients might be transferred to academic centers in the meantime. The presented study results are generalizable to the academic population, which could cover most PET/CTs performed for infection or inflammation.

So far, studies in children have focused on specific settings or symptoms, such as patients with known bacteremia or children with FUO, and are all retrospective. In 2016, Kouijzer et al. reported a high positive and negative predictive value of 71 and 100%, respectively, in a small cohort of 13 patients focused on metastatic infection with known bacteremia [17]. In a cohort of children with FUO (*n* = 31 with 28 FDG-PET/CT and 3 PET) PET/(CT) contributed to the diagnosis in 32% of cases, and in 58% of children with FUO and immune suppression (*n* = 12) [6]. Jasper et al. reported clinical helpfulness in 45% in 77 scans, including 22% by exclusion of diagnoses and 23% because PET/(CT) allowed targeted evaluation [7]. However, 47 scans were performed on stand-alone PET scanners without CT (with 40% helpful scans versus 53% helpful scans in PET scans with CT). A recent study including the largest cohort of pediatric patients with FUO thus far (*n* = 110) reported that in 68/110 (62%) patients, a definite cause of fever was identified, where in 53 of 68 (78%), this cause was detected by FDG-PET/CT (53 of a total of 110 scans, 48%) [8].

We found FDG-PET/CT to be helpful in 65% of diagnostic scans and in 71% of subjects a final diagnosis was established, of which a minority were reached without contribution of FDG-PET/CT. This is higher than in other studies on FUO, which can be explained by the fact that we included both children with a provisional diagnosis and children with a yet unknown diagnosis, and that scans were performed on hybrid PET/CT scanners, with a known higher diagnostic yield than stand-alone PET scanners. In children with a provisional diagnosis before scanning, 50% of negative scans contributed to a final diagnosis versus 22% in patients with a broad differential and no provisional diagnosis before scanning. Most studies on FUO only classify positive scans as contributory due to the nature of the diagnostic question in FUO, i.e., what is the source of fever [6,7,8,18,19]. Children with a broad differential and high diagnostic uncertainty before FDG-PET/CT are more comparable to FUO and only 2/9 negative scans in this category contributed to the final diagnosis. We chose this broader definition of helpfulness because both positive and negative scans can narrow down diagnostic possibilities. Besides contributing to establishing a diagnosis, negative FDG-PET/CT findings are associated with spontaneous regression of fever in adults [20]. Overall, the path towards a final diagnosis is a complex process of information gathering and interpretation, to generate a working diagnosis that enables management decisions and an explanation of the origin of the symptoms [21].

Although information obtained by FDG-PET/CT seemed to contribute to a final diagnosis or influence patient management decisions, it is difficult to reliably determine the added value of FDG-PET/CT results in the diagnostic process and subsequent management decisions in a retrospective study that relies on clinical records. After all, it is unknown how the diagnostic process and management would have evolved without FDG-PET/CT scanning. In addition, the likelihood that FDG-PET/CT would yield new information compared to previous diagnostics retrospectively sometimes seemed low, and the timing questionable. However, apparently diagnostic uncertainty prompted FDG-PET/CT imaging in the studied cases. More robust data could be generated in a randomized controlled trial. This is not feasible in our opinion, considering the broad implementation of FDG-PET/CT and the presence of (lower level) evidence that FDG-PET/CT is a valuable diagnostic tool.

A prospective observational study using a structured diagnostic protocol would be a feasible and acceptable alternative. This protocol would start with a multidisciplinary consultation with treating physicians and nuclear medicine physicians preceding FDG-PET/CT scanning, to discuss the clinical situation, and document the differential diagnosis and expected added value of FDG-PET/CT. FDG-PET/CT results would then be discussed in this team and interpreted in the light of the current clinical symptoms. Differential diagnosis after FDG-PET/CT scanning and perceived value of information generated by FDG-PET/CT are documented, along with associated management decisions. Follow-up is standardized, to test the accuracy of a potential final diagnosis and the role of FDG-PET/CT in establishing this diagnosis. Two goals could be met by the proposed study; collecting information on the added value of FDG-PET/CT and potential improvement of use and diagnostic yield of FDG-PET/CT by discussions between the nuclear medicine specialist and treating physician.

## 5. Conclusions and Recommendation

In this retrospective study on FDG-PET/CT use in children suspected of infection or inflammation, we found that FDG-PET/CT results were accurate and contributed to the final diagnosis or guided management in the majority of cases. High CRP was associated with positive findings, but low CRP did not preclude positive scans. Overall, FDG-PET/CT use is increasing and technological advances create promising possibilities. Prospective studies using a structured diagnostic protocol can generate more robust evidence on the diagnostic value of FDG-PET/CT. This includes interdisciplinary consultations between the treating physician, nuclear medicine physician and other healthcare providers with relevant expertise, which could further improve the indication, timing and interpretation of pediatric FDG-PET/CT.

## Figures and Tables

**Table 1:** Scan and patient characteristics.

	*n*	%	Median (IQR)
Characteristics			
Patients	59		
Age at first scan (yrs)			12 (5–15)
Female	37	(63)	
No of FDG-PET/CT scans	64		
diagnostic scans	50	(73)	
follow up scans	14	(27)	
>1 scan, No of patients	4		
Amsterdam University Medical Center	21	(33)	
University Medical Center Utrecht	17	(27)	
Erasmus University Medical Center	19	(30)	
Leiden University Medical Center	5	(8)	
Alrijne Hospital Leiderdorp	2	(3)	

**Table 2:** Characteristics of patients with diagnostic scans.

	*n*	%	Median (IQR)
Diagnostic scans and patients	48		
CRP in week before scan (mg/L)	46	(96)	44 (9–120)
Fever > 38.3 in week before scan	19	(40)	
**Preceding diagnostics**			
Serology (any lab test)	48	(100)	
**Radiology**			
Chest X ray	26	(54)	
Other radiological examination	14	(29)	
Ultrasonography	37	(77)	
MRI	17	(35)	
CT	11	(23)	
Bone Scintigraphy	1	(2)	
**Microbiology**			
Blood culture	34	(71)	
Urine culture	15	(31)	
Sputum/nose/throat culture	12	(25)	
Faeces culture/PCR testing	13	(27)	
Joint fluid culture	2	(4)	
Cerebrospinal fluid culture	6	(13)	
**Cytology/histology**	12	(25)	

## Supplementary Materials

The following are available online at https://www.mdpi.com/2075-4418/10/9/715/s1, Supplementary Table S1: Diagnostic question and diagnostic information and consequence of FDG-PET/CT performed for primary diagnosis (diagnostic scans); Supplementary Table S2: Clinical situation, result and consequence of FDG-PET/CT performed to monitor disease activity (follow-up scans).
